# Association between Parent and Child Dietary Sodium and Potassium Intakes: Aomori Prefectural Health and Nutrition Survey, 2016

**DOI:** 10.3390/nu11061414

**Published:** 2019-06-23

**Authors:** Tatsuya Koyama, Nobuo Yoshiike

**Affiliations:** Aomori University of Health and Welfare, 58-1 Mase-Hamadate, Aomori-shi 038-8505, Japan; n_yoshiike@auhw.ac.jp

**Keywords:** child, grandparent, parent, potassium, sodium

## Abstract

This study investigated the association between parent and child sodium and potassium intakes using data from the 2016 Aomori Prefectural Health and Nutrition Survey. We analyzed one day dietary record data of 103 mothers, 94 fathers, 51 children aged 1–3 years, 39 children aged 4–6 years, 91 children aged 7–14 years, and 56 children aged 15–19 years. We also examined the association of sodium and potassium intake between co-habiting grandparents and their grandchildren. After adjusting for covariates, the total daily sodium intake in mothers was positively associated with that in children for every age group. Potassium intakes by the mothers during breakfast and dinner were positively associated with those in children aged 1–3, 4–6, and 7–14 years. The associations in sodium and potassium intakes between fathers and children were weaker. In addition, these associations were similar to those between the sodium intakes of grandchildren and their grandparents. The association between mother and child sodium and potassium intakes at breakfast and dinner was related to the consumption of similar foods, which suggests the importance of home environment in influencing total dietary sodium and potassium intake in Japanese people.

## 1. Introduction

Excessive sodium and insufficient potassium intake can lead to hypertension and cardiovascular disease in adults [1,2]. Similarly, sodium and potassium intake are positively and inversely associated with blood pressure, respectively, in children [3,4]. Elevated blood pressure in childhood is a predisposing risk factor for hypertension in adulthood [5,6] and increases the risk of the early development of cardiovascular disease [7].

Although sodium intake in Japanese people has decreased since the 1950s, high sodium intake is still noted in this population [8]. Aomori prefecture is a region with high sodium intake in Japan. For example, according to the National Health and Nutrition Survey 2016 in Japan, the average sodium intake in Aomori prefecture was approximately 4450 mg/person/day, while that in Japan was approximately 4250 mg/day [9]. Aomori prefecture also has a high stroke mortality rate compared to other regions in Japan [10]. It is important to educate children in Japan about the importance of reduction of sodium intake, especially in Aomori prefecture.

While growing evidence suggests that dietary intake among children modestly resembles that of parents, the age of children may be associated with the parent–child dietary resemblance [11]. However, few studies have investigated the association between sodium and potassium intakes of parents and children in Japanese people [12]. While grandparents may influence the dietary intake of co-habiting grandchildren [13], little is known regarding whether the dietary intake among grandchildren resembles that of co-habiting grandparents. Most Japanese children attend schools on weekdays. Although Japanese children attend schools on weekdays and school meals may influence their total daily nutrient intake, parents can directly influence their dietary choices. [14].

Therefore, the aim of this study was to assess the association between parent and child sodium and potassium intakes stratified by eating occasion (total day, breakfast, and dinner). We also examined the association of sodium and potassium intake between co-habiting grandparents and their grandchildren.

## 2. Materials and Methods

### 2.1. Study Population

The present cross-sectional study was based on data from the 2016 Aomori Prefectural Health and Nutrition Survey. The full details of this survey are described elsewhere [15]. The survey was conducted in the same way as the National Health and Nutrition Survey in Japan [9]. Twelve census units in Aomori prefecture were randomly sampled as a survey area based on the population census. All non-institutionalized Japanese people aged ≥1 year living in these areas were invited to participate. The survey was conducted between October and November 2016. A total of 846 participants completed the dietary survey.

We included children 1–19 years of age with the complete 1 day dietary record data. Parents and grandparents were matched to the children by family relationship. After matching parents with their children, the final sample sizes were 197 parent-child (94 father–child and 103 mother–child) and 40 grandparent–grandchild pairs.

The survey was conducted according to the principles of the Declaration of Helsinki, and written informed consent was obtained from all individual subjects and their parents. The Aomori Prefectural Health and Nutrition Survey had stringent protocols and procedures to ensure confidentiality and to protect individual participants from identification. This survey was conducted according to the regulations of Aomori Prefecture. Additionally, the present secondary analysis was based on a contract research agreement and the public-use dataset consisting of only anonymized information. Thus, institutional review board approval was not required.

### 2.2. Assessment of Dietary Intake

Dietary intake data were collected using a 1 day semi-weighed household dietary record, the procedure of which has been described elsewhere [16]. Briefly, the participants and the main record-keeper were provided both written and verbal instructions by trained fieldworkers (registered dietitians) regarding the purpose of the dietary records and how to weigh and record the food items consumed by household members each day. When household members shared food from the same dish, the record-keeper was also asked to record the approximate proportions of food taken by each member so that the dietary intakes of each individual could be calculated. When weighing was not possible (e.g., eating out), the record-keeper was asked to record as much information as possible, including the portion size consumed and details of any leftovers. Children aged 7 to 14 years had lunch at school. School lunch was nutritionally managed based on the Criteria for Provision of School Lunch of the School lunch Act. School lunch was served so that salt intake was low and potassium intake was high by providing high vegetable and milk [17]. The recording day was freely selected by each household, except for Sundays, national holidays, and days with special events (e.g., weddings or funerals). Trained fieldworkers visited the household and checked the completeness of food recording and, when necessary, additional information was added. The estimated salt intake for each individual was calculated from the record of household food consumption and, for shared dishes or foods, approximate proportions consumed by each household member based on the Standard Table of Food Composition in Japan [18].

### 2.3. Statistical Analysis

We categorized the children into four age groups (1–3, 4–6, 7–14, and 15–19 years). All analyses were separated by eating occasion (daily total, breakfast, and dinner). We used descriptive statistics to summarize participants’ characteristics as means and standard deviations for continuous variables. Multiple linear regression was used to assess the association between child dietary intake (dependent variable) and parent dietary intake (independent variable). For children aged 1–3 and 4–6 years, we adjusted for parent age. For children aged 7–14 years, we adjusted for parent and child sex and age. For children aged 15–19 years, we adjusted for parent age and child sex.

The association between the energy-adjusted dietary intake of grandchildren and that of their grandparents was also examined using Spearman correlations. In this study, all grandparent-grandchild pairs were living together. Energy adjustments were made using density methods. The energy-adjusted total daily intakes of sodium and potassium were determined by dividing the total daily sodium and potassium intakes by the total daily energy intake. Since there were only three pairs of grandparent–grandchildren aged 4–6 years, we did not show these data.

All statistical analyses were performed using SPSS version 24.0 (IBM Corporation, Tokyo, Japan). All reported p-values were two-tailed, and *p*-values <0.05 were considered statistically significant.

## 3. Results

On average, the sodium intake at breakfast accounted for 19–29%, while dinner accounted for 31–50% of the intake (Table 1). Similarly, on average, the potassium intake at breakfast and dinner accounted for 20–29% and 29–52% of the intake.

A 1 mg/day increase in the mother’s sodium intake was associated with a 0.4–1.4 mg/day increase in child’s salt intake (Table 2). This association was observed for both breakfast and dinner. The potassium intakes at breakfast and dinner for mothers were positively associated with those for children in all four age groups, except for dinner in children aged 15–19 years. Compared with that of mother–child, the father–child association appeared to be weaker.

There were significant positive correlations in energy-adjusted total daily sodium intake between grandparents and grandchildren aged 1–3 years (Table 3). There were significant positive correlations in energy-adjusted total daily intakes of sodium and potassium between grandparents and grandchildren aged 7–14 years. Finally, there were significant positive correlations in energy-adjusted intakes of sodium and potassium during breakfast and dinner between grandparents and grandchildren aged 15–19 years. 

## 4. Discussion

Our results demonstrate that the sodium intake of parents, especially mothers, was positively associated with that of children for most age groups. In addition, these results were consistent with the association between the sodium intakes of grandchildren and their grandparents. The potassium intakes of the mothers at breakfast and dinner were positively associated with those of their children. These findings are similar to those of previous studies. The Framingham Heart Study (FHS) [19] and the Salt and Other Nutrient Intakes in Children (SONIC) study [20] reported a correlation between mother and child’s sodium intakes but not in father’s sodium intake or in either parent’s potassium intakes. Mothers usually devote more time to childcare [21]. Women are more likely to have the primary responsibility for meal planning, shopping, and preparation [22,23]. Moreover, long working hours among fathers are positively associated with take-out meals, missed family meals, and eating while working [24]. The increased association between mother and children is likely explained by these observations. Family meals are positively associated with a parent–child association [25]. The association between grandparents and their grandchildren may be explained by family meals.

Service et al. explained the different association between sodium and potassium intakes by a similarity in dietary sources [19]. The major sources of sodium in children’s and adults’ diets were similar, while the dietary sources of potassium differed between adults and children. However, to our knowledge, there are no data about dietary sources of sodium and potassium in Japanese children. Our results showed that the proportion of potassium intake at lunch was larger than that of sodium intake at lunch for children. This may explain the lack of association between the total daily potassium intakes in parents and children.

Taste preferences for sodium are determined by exposure to salty foods; both short-term and lifelong exposures to higher sodium intake may lead to a preference for higher-sodium foods [26,27,28,29]. Given that taste and dietary preferences appear to develop and track over life, it is important that childhood sodium intake is not excessive [30]. Parents may play an important role in the reduction of sodium intake of children by reducing seasoning during cooking and choosing lower-sodium manufactured products, thereby reducing the threshold of salt taste early in life.

This study had several limitations. First, our results may not be generalizable to the Japanese population because the number of participants, especially children aged 1–3 and 4–6 years, was small, and the study was conducted in only one prefecture. Sodium intake in Aomori prefecture is higher than the national average in Japan [9]. The prevalence of obesity in children is also higher in Aomori prefecture [31]. Although this study was done in one area of Japan, it is an important area in considering Japanese health problems. Moreover, sodium and potassium intakes were estimated using a one day dietary record. Considering that dietary intake varies day-to-day among free-living individuals, the dietary patterns revealed in this study from one day weighed household dietary record do not likely represent the usual patterns of the individual respondents. In this study, the estimated intakes of sodium and potassium did not compare to 24 h urinary and potassium excretion. Therefore, the validity of the household dietary records was unknown.

## 5. Conclusions

The results of this study indicate an association between mother and child sodium intakes in total daily and potassium intake at breakfast and dinner in Japanese people. This finding suggests that family-based interventions are more likely to have greater effects in reducing sodium intake and increasing potassium intake in families with children, which will assist in reducing the risk of cardiovascular disease later in life.

## Figures and Tables

**Table 1:** Dietary intakes of children, mothers, fathers, and grandparents.

Dietary Intakes	Children								Mothers (*n* = 103)		Fathers (*n* = 94)		Grandparents (*n* = 40)	
	1–3 Years (*n* = 51)		4–6 Years (*n* = 39)		7–14 Years (*n* = 91)		15–19 Years (*n* = 56)							
Men	24	47%	17	44%	44	48%	25	45%	103	100%	0	0%	16	40%
Age (years)	1.8	(0.9)	5.0	(0.7)	10.5	(2.4)	16.4	(1.3)	38.9	(7.1)	40.2	(7.8)	71.2	(8.1)
Energy (kcal)														
Daily total	1083	(209)	1356	(239)	1849	(362)	2128	(550)	1641	(402)	2094	(525)	1880	(583)
Breakfast	264	(72)	348	(127)	459	(150)	466	(244)	399	(220)	417	(265)	499	(211)
Dinner	335	(134)	446	(201)	645	(234)	824	(199)	670	(236)	910	(277)	815	(333)
Sodium (mg)														
Daily total	2002	(871)	2669	(966)	3519	(1078)	3765	(947)	3631	(1544)	3941	(1603)	4119	(1529)
Breakfast	534	(334)	615	(466)	918	(602)	785	(515)	940	(854)	753	(640)	1176	(1050)
Dinner	630	(539)	1080	(687)	1284	(595)	1891	(825)	1574	(901)	1771	(881)	1916	(745)
Potassium (mg)														
Daily total	1400	(517)	1471	(398)	2085	(651)	2062	(558)	1935	(652)	1949	(750)	2346	(1169)
Breakfast	323	(190)	297	(160)	458	(269)	482	(319)	473	(332)	407	(326)	682	(488)
Dinner	411	(249)	486	(240)	803	(412)	892	(376)	844	(362)	1014	(410)	1007	(501)

Values are shown as number (%) or mean (standard deviation).

**Table 2:** Association between parent–child sodium and potassium intakes.

Dietary Intake	Child	Eating Occasion	Mother				Father			
			*n*	*β*	(95% CI)	*P*	*n*	*β*	(95% CI)	*P*
Sodium (mg)	Child (1–3 years)	Daily total	21	1.35	(0.67–2.04)	0.001	21	0.19	(−0.11–0.48)	0.198
		Breakfast	21	1.81	(0.78–2.83)	0.002	21	0.61	(0.30–0.91)	0.001
		Dinner	21	0.72	(0.19–1.24)	0.010	21	0.09	(−0.23–0.23)	0.412
	Child (4–6 years)	Daily total	20	0.39	(0.16–0.63)	0.003	16	0.39	(0.10–0.68)	0.012
		Breakfast	20	0.22	(0.01–0.43)	0.044	16	0.50	(0.27–0.73)	<0.001
		Dinner	20	0.73	(0.49–0.97)	<0.001	16	0.85	(0.42–1.28)	0.001
	Child (7–14 years)	Daily total	40	0.60	(0.40–0.80)	<0.001	36	0.29	(0.04–0.55)	0.024
		Breakfast	40	0.42	(0.15–0.70)	0.003	36	0.28	(−0.08–0.63)	0.120
		Dinner	40	0.72	(0.48–0.95)	<0.001	36	0.30	(0.12–0.48)	0.002
	Child (15–19 years)	Daily total	22	0.39	(0.09–0.69)	0.014	21	0.15	(−0.15–0.45)	0.317
		Breakfast	22	0.40	(0.08–0.72)	0.017	21	0.03	(−0.35–0.41)	0.852
		Dinner	22	0.61	(0.48–0.74)	<0.001	21	0.74	(0.44–1.03)	<0.001
Potassium (mg)	Child (1–3 years)	Daily total	21	0.15	(−0.57–0.86)	0.674	21	−0.12	(−0.41–0.17)	0.396
		Breakfast	21	1.14	(0.33–1.94)	0.008	21	0.43	(0.27–0.60)	<0.001
		Dinner	21	0.98	(0.53–1.43)	<0.001	21	0.42	(0.08–0.77)	0.018
	Child (4–6 years)	Daily total	20	0.89	(−0.22–0.40)	0.546	16	−0.35	(−0.81–0.11)	0.123
		Breakfast	20	0.39	(0.13–0.85)	0.006	16	0.25	(−0.10–0.59)	0.144
		Dinner	20	0.53	(0.08–0.97)	0.023	16	−0.20	(−0.85–0.46)	0.532
	Child (7–14 years)	Daily total	40	0.41	(0.06–0.77)	0.023	36	0.24	(−0.03–0.52)	0.080
		Breakfast	40	0.43	(0.06–0.80)	0.024	36	0.06	(−0.29–0.42)	0.721
		Dinner	40	0.68	(0.30–1.05)	0.001	36	0.11	(−0.17–0.40)	0.422
	Child (15–19 years)	Daily total	22	0.41	(−0.03–0.86)	0.066	21	0.21	(−0.16–0.58)	0.241
		Breakfast	22	−0.18	(−1.04–0.68)	0.863	21	0.53	(−0.36–0.46)	0.788
		Dinner	22	0.49	(0.20–0.79)	0.003	21	0.65	(0.20–1.11)	0.007

Child (1–3 years), child (4–6 years): adjusted for age of parent. Child (7–14 years): adjusted for age of parent, sex, and age of child. Child (15–19 years): adjusted for age of parent, and sex of child.

**Table 3:** Grandparent–grandchild correlations in sodium and potassium intake.

Dietary Intakes	Total Day			Breakfast			Dinner		
	*ρ*	(95% CI)	*P*	*ρ*	(95% CI)	*P*	*ρ*	(95% CI)	*P*
Sodium (mg/1000 kcal)									
Grandchild (1–3 years) (*n* = 9)	0.713	(0.093–0.935)	0.031	0.558	(−0.169–0.892)	0.118	0.112	(−0.596–0.722)	0.775
Grandchild (4–6 years) (*n* = 15)	0.757	(0.400–0.915)	0.001	0.418	(−0.120–0.766)	0.121	0.520	(0.011–0.815)	0.047
Grandchild (15–19 years) (*n* = 14)	0.415	(−0.148–0.775)	0.159	0.606	(0.111–0.860)	0.028	0.613	(0.122–0.863)	0.026
Potassium (mg/1000 kcal)									
Grandchild (1–3 years) (*n* = 9)	0.292	(−0.462–0.801)	0.446	0.412	(−0.347–0.845)	0.270	0.421	(−0.337–0.848)	0.260
Grandchild (4–6 years) (*n* = 15)	0.880	(0.670–0.960)	<0.001	0.478	(−0.045–0.795)	0.072	0.955	(0.867–0.985)	<0.001
Grandchild (15–19 years) (*n* = 14)	0.188	(−0.381–0.653)	0.539	0.793	(0.453–0.932)	0.001	0.643	(0.171–0.875)	0.018

*ρ*, Spearman’s correlation coefficient. Since number of pairs of grandparent–grandchildren aged 4–6 years was three, we did not show data.
